# Supplementary material for: Disentangling the consequences of type 2 diabetes on targeted metabolite profiles using causal inference and interaction QTL analyses
Source: PLoS Genet. 2024 Dec 3;20(12):e1011346. doi: 10.1371/journal.pgen.1011346 (PMC11642953; doi:10.1371/journal.pgen.1011346)
Supplement: S2 Appendix — Fig A: Workflow to define prevalent T2D cases at T0, when metabolite data have been measured. Fig B: Comparison of betas and p-values with Smith et al. 2023 for the reverse MR on the first set of metabolites [18]. Fig C: Manhattan plot of the interaction QTL analysis for the four metabolites significant and replicated in the EstBB. Fig D: Genomic regions around the most significant variants from the interaction QTL analyses, obtained from https://genome.ucsc.edu/. (DOCX) [file pgen.1011346.s002.docx]

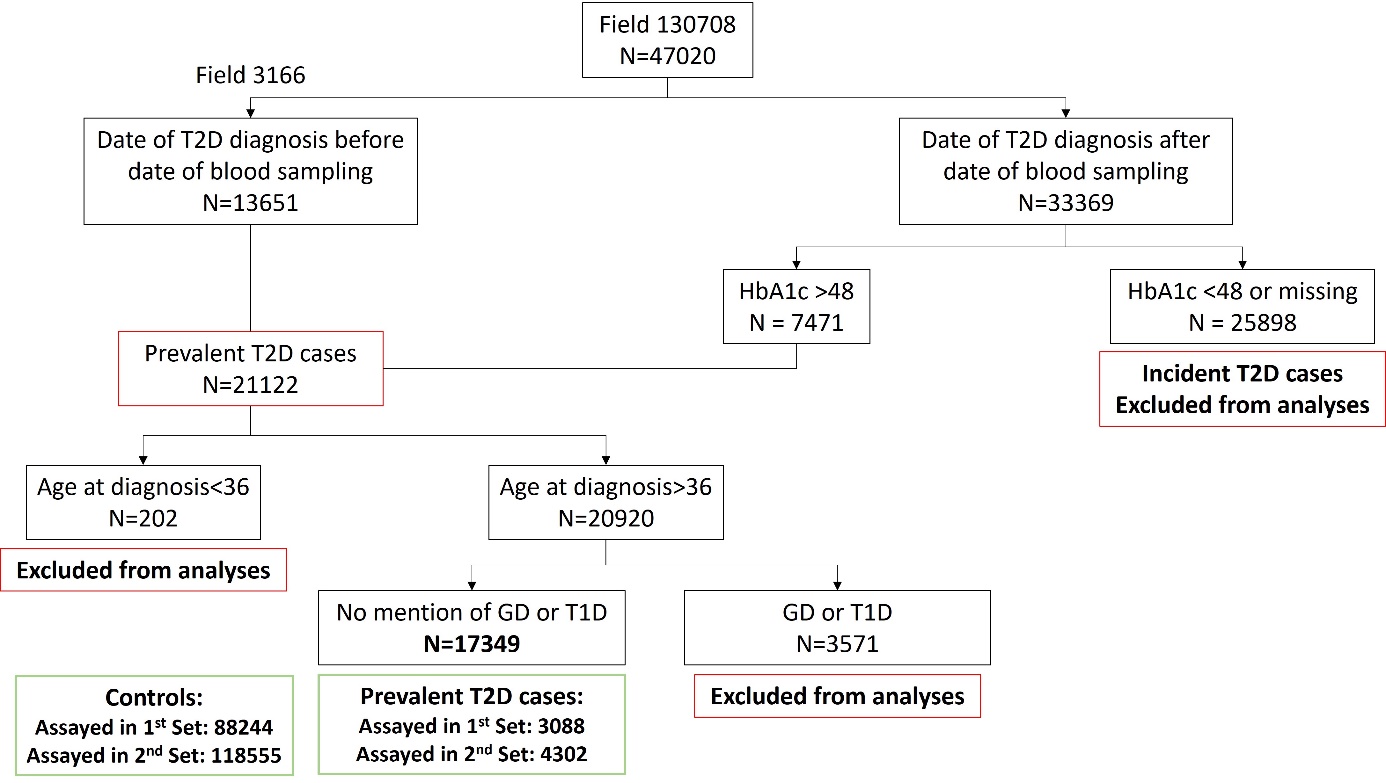


Fig A: Workflow to define prevalent T2D cases at T0, when metabolite data have been measured.


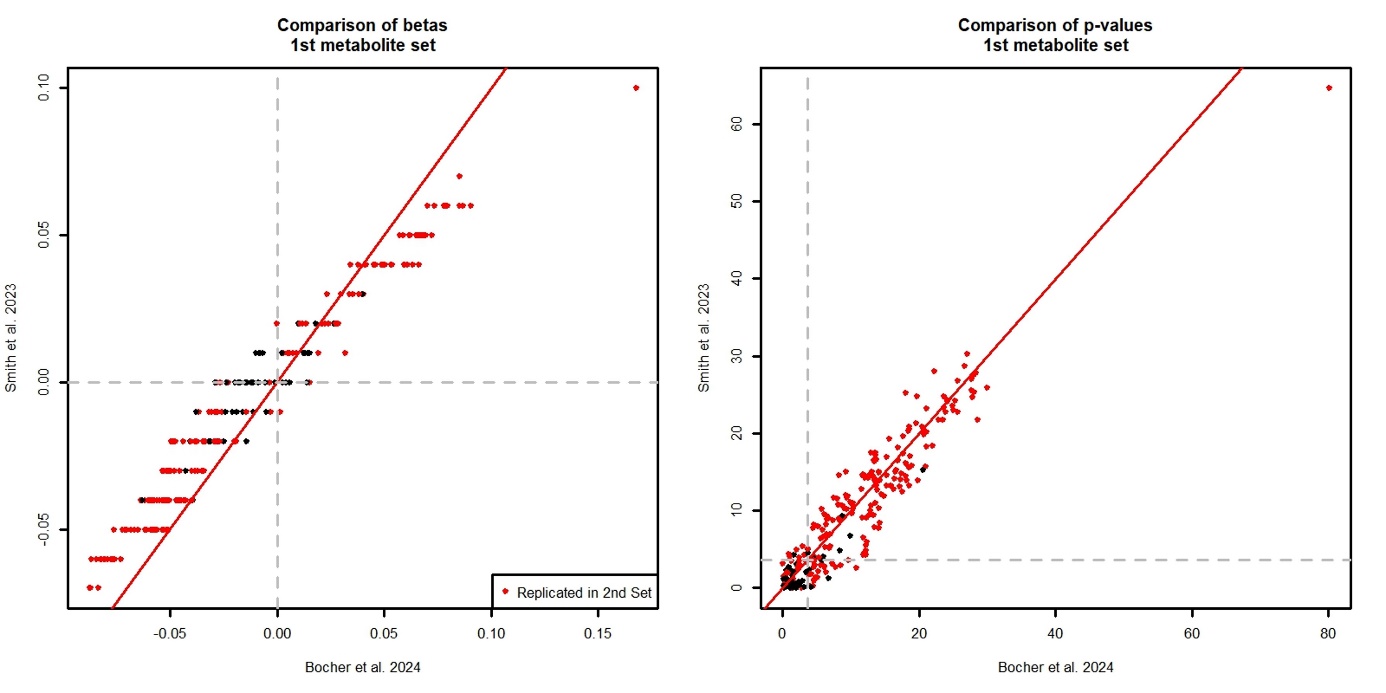


Fig B: Comparison of betas and p-values with Smith et al.[1] for the reverse MR on the first set of metabolites.


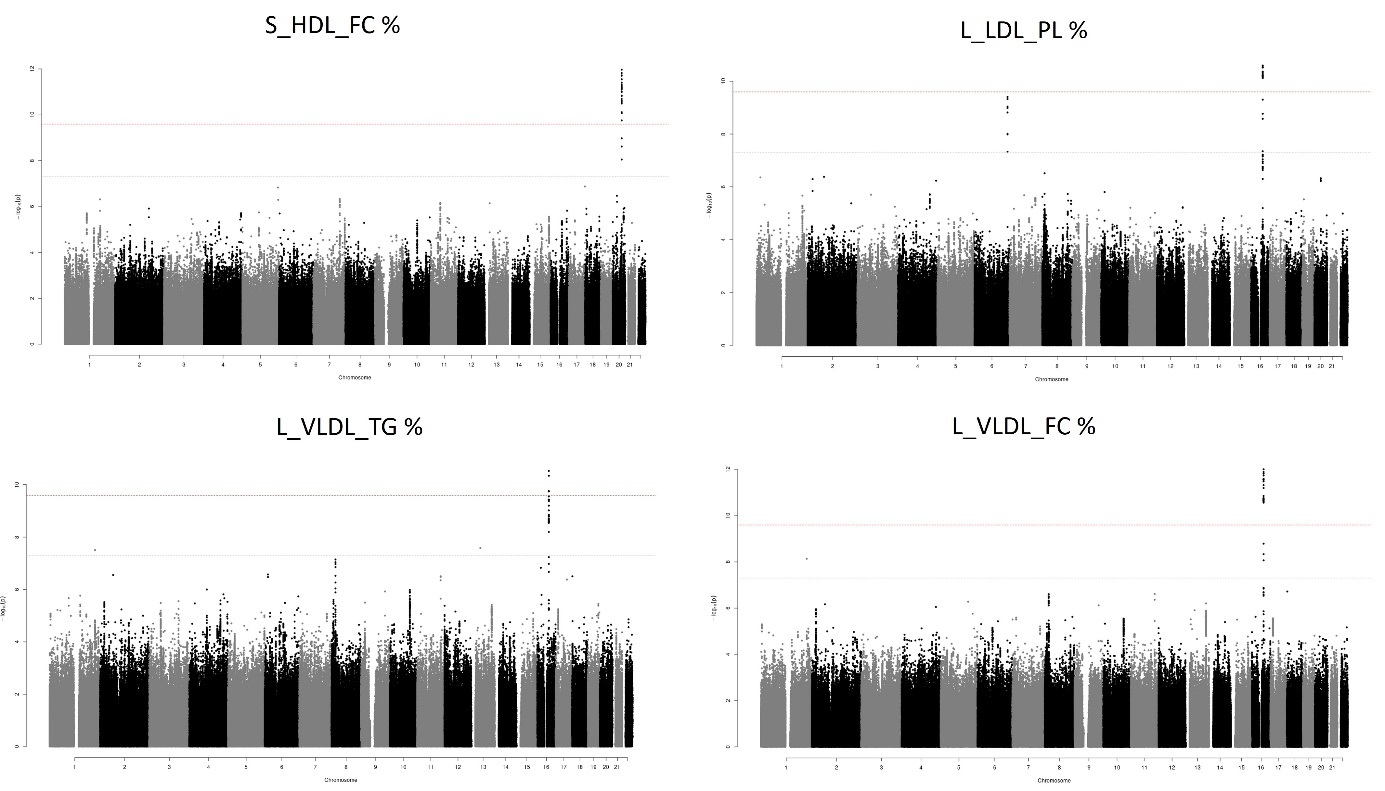


Fig C: Manhattan plot of the interaction QTL analysis for the four metabolites significant and replicated in the EstBB.


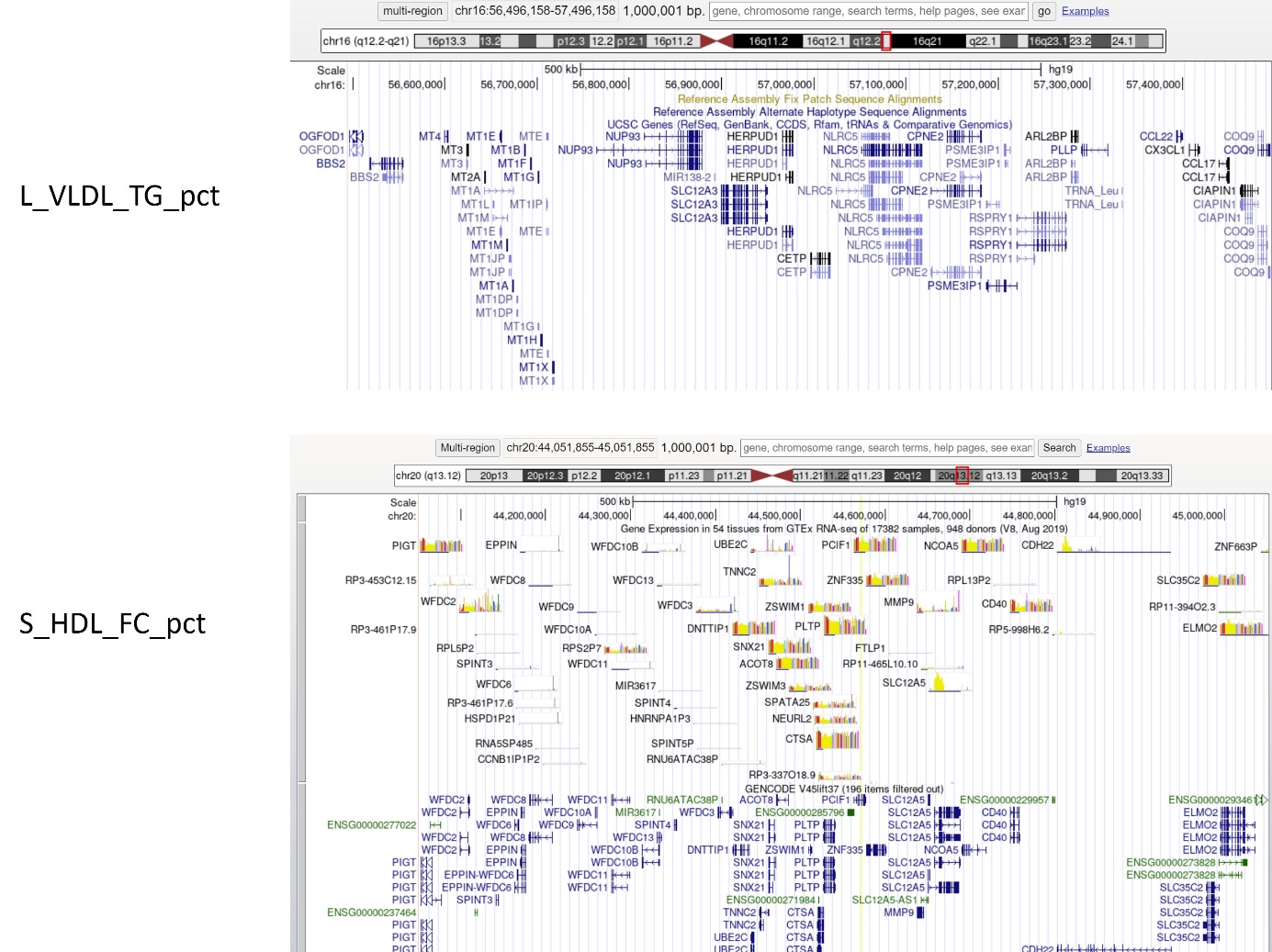


Fig D: Genomic regions around the most significant variants from the interaction QTL analyses, obtained from https://genome.ucsc.edu/

References

1. Smith ML, Bull CJ, Holmes MV, Davey Smith G, Sanderson E, Anderson EL, et al. Distinct metabolic features of genetic liability to type 2 diabetes and coronary artery disease: a reverse Mendelian randomization study. EBioMedicine. 2023 Apr;90:104503.
