# Supplementary material for: Disentangling the consequences of type 2 diabetes on targeted metabolite profiles using causal inference and interaction QTL analyses
Source: PLoS Genet. 2024 Dec 3;20(12):e1011346. doi: 10.1371/journal.pgen.1011346 (PMC11642953; doi:10.1371/journal.pgen.1011346)
Supplement: Attachment — Submitted filename: UKB_Metabolites_T2D_Reviewers comments-V2.docx [file pgen.1011346.s004.docx]

We thank the reviewers for all their comments on the manuscript and for giving us the opportunity to submit a revised version. A point-by-point response is provided below.

Reviewer #1: In the manuscript entitled “Insights into the Metabolic Consequences of Type 2 Diabetes,” the authors aim to link diabetes, metabolites, and the genetic component. This study primarily replicates previous findings rather than reporting new ones. The following points could be considered to improve the manuscript's quality:

**1. In the abstract the authors are using metabolomics measurements from Nightingale platform which is limited in terms of the number of measured metabolites and is more lipoproteins oriented. Thus, statement such as: “Overall, this work provides a map of the metabolic consequences” might be misleading especially for people who are not familiar with the field of metabolomics.**

We thank the reviewer for this comment. We have now changed the abstract to specify the number of metabolites and the panel used in this study:

*“Circulating metabolite levels have been associated with type 2 diabetes (T2D), but the extent to which T2D affects metabolite levels and their genetic regulation remains to be elucidated. In this study, we investigate the interplay between genetics, metabolomics, and T2D risk in the UK Biobank dataset using the Nightingale panel composed of 249 metabolites, 92% of which correspond to lipids (HDL, IDL, LDL, VLDL) and lipoproteins. By integrating these data with large-scale T2D GWAS from the DIAMANTE meta-analysis through Mendelian randomization analyses, we find 79 metabolites with a causal association to T2D, all spanning lipid-related classes except for Glucose and Tyrosine. Twice as many metabolites are causally affected by T2D liability, spanning almost all tested classes, including branched-chain amino acids. Secondly, using an interaction quantitative trait locus (QTL) analysis, we describe four metabolites consistently replicated in an independent dataset from the Estonian Biobank, for which genetic loci in two different genomic regions show attenuated regulation in T2D cases compared to controls. The significant variants from the interaction QTL analysis are significant QTLs for the corresponding metabolites in the general population but are not associated with T2D risk, pointing towards consequences of T2D on the genetic regulation of metabolite levels. Finally, through differential level analyses, we find 165 metabolites associated with microvascular, macrovascular, or both types of T2D complications, with only a few discriminating between complication classes. Of the 165 metabolites, 40 are not causally linked to T2D in either direction, suggesting biological mechanisms specific to the occurrence of complications. Overall, this work provides a map of the consequences of T2D on Nightingale targeted metabolite levels and on their genetic regulation, enabling a better understanding of the T2D trajectory leading to complications.”*

**2. In the abstract the authors are reporting on 165 metabolites identified to be associated with T2D complication. To bring the reader perspective, it would be important to mention out of how many.**

The number of metabolites assessed by the Nightingale panel has now been added at the beginning of the abstract in line 23.

**3. In the abstract the authors are writing that mostly spanning lipid-related classes but the method is measuring mainly lipids and lipoproteins so it is not surprising that such observation was made. It would be suggested to differently address this.**

In the abstract, in addition to adding the number of metabolites in the Nightingale panel, we have also specified the percentage of lipids and lipoproteins present in this panel. We have also improved the description of results by noting the significant metabolite classes in the abstract.

**4. In the introduction short references to the Nightingale platform briefly describing the number of molecule measured and nature of those molecules mentioning that mainly lipoproteins are measured would be beneficial for the reader. Including this information in the methods only is not sufficient.**

We thank the reviewer for this useful suggestion which will help the reader in better understanding the metabolite panel used. We have added more details on this panel, including the metabolite classes, in lines 84-88:

*“[…] For this, we considered profiles of 249 metabolites from the UKBB cohort^1^ assessed through the Nightingale panel, mostly composed of lipids and lipoproteins classes (around 92% of metabolites), and covering additional classes such as amino acids (10 metabolites), ketone bodies (4 metabolites), glycolysis-related metabolites (4 metabolites), and markers of inflammation (1 metabolite). These levels were characterized for almost 275,000 participants through two releases, which were here considered for discovery (n=117,967 individuals) and replication (n=156,385 individuals), or meta-analyzed when statistically appropriate. […]”*

**5. For the methods. Although the Mendelian randomization is extensively described following points could be addressed:**

**A) Specify Software Versions: Include the specific versions of the software and libraries used, e.g., "We used the MendelianRandomization library (versionX) and the TwoSampleMR library (versionX)."**

We have now added software version information in lines 187-188.

**B) Although the authors describe that they are using Borges summery statistics. The clear specify the sources of the datasets and context on why these sources were chosen is missing.**

We have now added information on the mQTL map from Borges et al. in lines 166-168:

*“[…] For the first release dataset, we used mQTL summary statistics from Borges et al.^2^, which correspond to the QTL map of the 249 metabolites from the first data release that has been generated in the UK Biobank. For the second release, we performed a mQTL analysis using the REGENIE software with the --qt option considering the INT metabolite levels as the phenotype (version 2.2.4)^3^. […]”*

**C) Same for REGENIE software more clarity regarding this should be also added.**

We thank the reviewer for this suggestion. As the software version was already reported in line 214, we have now also specified in the text the options that were used to run the command to perform the mQTL (lines 168-169) and interaction mQTL analyses (line 214).

**D) It would be recommended to describe instrument selection criteria in greater detail by including significance thresholds and LD clumping parameters.**

The significance thresholds and LD clumping parameters used for the selection of IVs are described in lines 173-176. We have reworded text in lines 173-181 to make it clearer that IVs were selected from independent variants obtained after clumping:

*“[…] Instrumental variables (IVs) were selected from independent variants defined through LD-based clumping using plink^4^ with the following parameters: R^2^<0.001 in windows of 10Mb, a p-value threshold of 2.54x10^‑10^ for the metabolite levels and of 5x10^-8^ for T2D. The threshold of 2.54x10^‑10^ is a genome-wide threshold corrected by the number of effective tests^5^, estimated at 197, which enables to correct the analyses for multiple testing while considering the correlation between the metabolites. For each of these independent variants, the strength of association with the exposure was determined using F-statistics. For the first released dataset and T2D summary statistics, the F-statistic was estimated using* $\frac{\beta^{2}}{{se}^{2}}$*, where beta is the effect estimate and se its corresponding standard error. […]”*

**E) The potential confounders could be better addressed by stating how potential confounders are handled or filtered out during the IV selection process.**

Potential confounders were not considered when selecting the IVs in order to fully represent the genetic architecture of the exposure. However, given this comment and an additional comment from another reviewer, we have performed sensitivity analysis within the UKBB to limit the potential bias due to statin medication, which is both linked to T2D risk and LDL profiles and could therefore be a potential confounder. For this, we performed one-sample MR restricted to individuals with no mention of statin medication as MVMR approaches were not possible due to the lack of GWAS investigating statin medication. We observed concordant MR results with this analysis, i.e. all the metabolites show concordant direction of effects with the main analyses and most of them, including all LDL-related metabolites, retain FDR-significance. These analyses are now described in lines 201-210 and lines 310-319.

*Methods section: “Statin medication, prescribed to lower LDL-related levels, is known to have an impact on T2D^6,7^ and could therefore bias the MR results. To limit the impact of statin medication in our MR analyses, we therefore performed one-sample MR in the forward direction restricted to UKBB participants not taking any statin medication. For this, we removed individuals taking medication with an ATC code starting with C10A or C10B corresponding to ‘lipid lowering agents’. We performed this sensitivity analysis in the second release set for the significant metabolites in the forward direction using the ivreg package (version 0.6-1) (96,380 individuals with 1,044 T2D cases and 95,336 controls). We used the same IVs as in the main MR analyses in the second release set and recomputed the F-statistics on the set of individuals not under statin medication to select only IVs with F>10. As for the other sensitivity analyses, we then compared the directions of effect.”*

*Results section: “Statin medication, used to lower LDL-C levels, is known to be associated with an increased risk of developing T2D^6,7^. To avoid potential bias in the MR analyses, we performed an additional sensitivity analysis in the forward direction in the second released metabolite restricted to individuals not taking statin medication through one-sample MR within the UKBB. We observed the same negative direction of effect for all LDL-related metabolites, including LDL-C, which retained FDR significance (S4 Table). The magnitude of the effect was lower, which could be due to the lower statistical power of one-sample MR compared to two-sample MR using large T2D summary statistics. These results suggest that the causal effect of LDL-related metabolites is independent of statin medication, in line with previous studies showing that, while statins have a causal effect on LDL-C levels, there is no evidence for a direct causal impact on T2D risk^8,9^.”*

Additionally, we acknowledge the need of further study using multivariate models to investigate potential biological pathways mediating causal effects between T2D and metabolite profiles in lines 546-550:

*“[…] In this study, we have here addressed the potential confounding effect of lipid-lowering medications in our MR analyses using restricted one-sample MR. It would however be interesting to use multivariate models to include variables such as BMI to better understand the biological pathways mediating causal relationships between T2D and metabolite profiles.”*

**F) The process of harmonizing exposure and outcome data before analysis should be better described.**

We thank the reviewer for this suggestion. More details on the harmonizing steps between the exposure and the outcome data have now been added in lines 186-188: *“Data were harmonized using the function harmonise_data() with default parameters from the R package TwoSampleMR (version 0.5-6)^10^, which was further used to run the MR analyses.”*

**G) More details on the sensitivity analyses performed to check the robustness of the results should be included.**

The sensitivity analyses performed are listed in lines 191-192, and additional details have now been provided regarding the choice of the sensitivity analyses, especially MR-Egger and the Steiger filtering, in lines 193-196:

*“These sensitivity analyses enable to evaluate causation under different MR assumptions, such as MR-Egger being robust to pleiotropy, or Steiger-filtered IVW removing IVs more strongly associated with the outcome than with the exposure to limit reverse causation effects.”*

For clarification, we also specified the sensitivity methods used at the beginning of the results section in lines 268-272:

*“The upset plot in Fig 1 represents the causal relationships between T2D and metabolites from the bi-directional MR that were significant in the first release dataset and replicated in the second release dataset, and which passed all our sensitivity criteria. This includes no significant pleiotropy and no significant heterogeneity (assessed at 5%), and concordant directions of effect across six sensitivity methods (MR Egger, weighted median, simple mode, weighted mode, IVW fixed effect, IVW random effect and Steiger filtered IVW). […]”*

**H) It would be recommended to provide details on how heterogeneity and pleiotropy were assessed and mention the specific statistics used.**

As indicated in lines 197-198, heterogeneity was assessed using the Q-statistics, and pleiotropy using the MR-Egger intercept p-value. We have added the information on the significance threshold in line 198, which corresponds to a 5% significance threshold. For the sensitivity methods, we have further incorporated this information at the beginning of the results section in line 271-272.

**I) If applicable, explain how the analysis is performed when only a single genetic variant is available**.

All metabolites had more than one selected IV (ranging from 5 to 75 IVs for the first set of metabolites using the summary statistics from Borges et al., and from 4 to 90 IVs for the second set of metabolites based on our mQTL analysis). This information has now been added in the text in lines 183-185 for clarification:

*“[…] Only IVs with an F-statistic larger than 10 were retained, which corresponds to a total of 5 to 75 IVs when using the mQTL statistics from Borges et al. based on the first metabolite release, and 4 to 90 IVs when using the results from our mQTL analysis performed on the second metabolite release.”*

**6. Given that the study was conducted in two big cohort, the potential clinical relevance of the discovery could be addressed in the discussion.**

We thank the reviewer for this suggestion. We have discussed the role of one of the interaction signals as being a regulator of CETP, a target of lipid-lowering drugs, in lines 510-512. We have now included interpretation of the second interaction signal, which is a QTL variant of the *PLTL* gene, and how these analyses can help in further understanding lipid profiles in T2D patients in lines 522-527:

*“[…] On the other hand, variants in the interaction region for S_HDL_FC_pct are QTL variants of the PLTP gene, which is involved in the transfer of phospholipids from triglycerides to HDL and might be involved in cholesterol metabolism. Investigating the differential genetic regulation of metabolite profiles in T2D patients might therefore help in better understanding how lipid profiles are affected upon T2D, a question still debated across studies^11^.”*

Reviewer #2: The authors conducted an extensive bi-directional Mendelian randomization (MR) study investigating the relationship between circulating metabolite levels and type 2 diabetes (T2D) using metabolomics data from the UK Biobank along with T2D summary statistics. Additionally, they performed an interaction QTL analysis within the UK Biobank on metabolite levels, using diabetes as an interaction variable, and examined complications.

**Study Design and Results:**

**The study design of the MR is generally well-articulated, and the results are clearly presented. However, I have a few remarks.**

**Firstly, the authors mentioned using existing summary statistics from Borges et al., but the reference cited corresponds to a different study by this author. I assume the correct reference is:**

**- Borges MC, et al. Role of circulating polyunsaturated fatty acids on cardiovascular diseases risk: analysis using Mendelian randomization and fatty acid genetic association data from over 114,000 UK Biobank participants. BMC Med. 2022 Jun 13;20(1):210. PMID: 35692035.**

**Please confirm this reference.**

We are grateful to the reviewer for spotting this error, and we have modified the reference accordingly.

**Figure 1 indicates that all identified associations in the reverse and forward MR were previously reported by Julkunen. While the lack of novelty in the reported numbers is not a major issue, given that the authors aim to disentangle the causal direction of association (metabolite → diabetes and diabetes → metabolite), there are concerns regarding the interpretation of the data in a causal context:**

**1. Strong causal effect of low LDL on increased T2D risk.**

**2. Diabetes causally lowering many cholesterol-related metabolites.**

**The authors explain the first observation by referencing studies that suggest low LDL is associated with increased diabetes risk, but they fail to consider a possible bias introduced by statins, which are known to influence both metabolite levels (LDL) and the outcome (diabetes). Instead, the authors justify this observation using other studies:**

**- The first study shows an inverse association between LDL GRS and diabetes in individuals not on lipid-lowering medications. However, the 31 SNP LDL GRS included HMGCR, the target of statins, implying a genetically induced statin-like effect.**

**- The second study is an observational study with Vanderbilt EHR data, which acknowledges potential exposure misclassification due to missing statin exposure data across multiple healthcare providers, despite conducting a sensitivity analysis.**

**- The third study indicates that familial hypercholesterolemia is associated with lower diabetes risk, which is not directly comparable.**

**The lack of discussion on the potential impact of statin-induced diabetes is concerning.**

We thank the reviewer for this comment and acknowledge that the discussion around the potential impact of statin-induced diabetes is necessary and relevant. As the third cited study is indeed not directly comparable, we have decided to remove it. In the first version of the manuscript, we had performed adjustment on lipid-lowering medication only for the interaction QTL analysis and the differential analysis with complications (details on this adjustment have been added in lines 227-228). However, we could not directly adjust the MR analysis using multivariate analyses as there are no GWAS directly representing statin medication. To answer this issue in the revised version of the manuscript, we have rather performed a one-sample MR analysis within the UKBB in the second release metabolite set in the forward direction (metabolites 🡪 T2D) restricted to individuals showing no record of taking statin medication. We have not performed this sensitivity analysis in the other MR direction as the statistical power would be much lower than when using the DIAMANTE summary statistics to select IVs for T2D exposure. We observed the same direction of effect for all significant metabolites between this one-sample MR analysis and the main analysis, with the LDL-related metabolites remaining significant at an FDR-corrected 5% threshold (results incorporated in Supplementary Table 4). This suggests that the causal effect of lower LDL levels on higher risk of T2D is independent of lipid-lowering medication. Methodological details have been added in lines 201-210, the results have been integrated in lines 310-319 in a paragraph which we have rewritten, and further discussed in lines 486-488:

*Methods section: “Statin medication, prescribed to lower LDL-related levels, is known to have an impact on T2D^6,7^ and could therefore bias the MR results. To limit the impact of statin medication in our MR analyses, we therefore performed one-sample MR in the forward direction restricted to UKBB participants not taking any statin medication. For this, we removed individuals taking medication with an ATC code starting with C10A or C10B corresponding to ‘lipid lowering agents’. We performed this sensitivity analysis in the second release set for the significant metabolites in the forward direction using the ivreg package (version 0.6-1) (96,380 individuals with 1,044 T2D cases and 95,336 controls). We used the same IVs as in the main MR analyses in the second release set and recomputed the F-statistics on the set of individuals not under statin medication to select only IVs with F>10. As for the other sensitivity analyses, we then compared the directions of effect.”*

*Results section: “Statin medication, used to lower LDL-C levels, is known to be associated with an increased risk of developing T2D^6,7^. To avoid potential bias in the MR analyses, we performed an additional sensitivity analysis in the forward direction in the second released metabolite restricted to individuals not taking statin medication through one-sample MR within the UKBB. We observed the same negative direction of effect for all LDL-related metabolites, including LDL-C, which retained FDR significance (S4 Table). The magnitude of the effect was lower, which could be due to the lower statistical power of one-sample MR compared to two-sample MR using large T2D summary statistics. These results suggest that the causal effect of LDL-related metabolites is independent of statin medication, in line with previous studies showing that, while statins have a causal effect on LDL-C levels, there is no evidence for a direct causal impact on T2D risk^8,9^.”*

*Discussion section: “It is to note that in our one-sample forward MR sensitivity analysis restricted to UKBB participants not under statin medication, LDL-related metabolites retained significance with the same direction of effect as in the two-sample MR analyses.”*

**Regarding the second observation, diabetes primarily lowering all types of cholesterol-related metabolites raises questions about the validity of the causal associations presented. Generally, diabetes leads to dyslipidemia rather than lowering cholesterol levels.**

We thank the reviewer for this question. For the LDL-related metabolites, the effects observed in our study are concordant with what was previously observed in the UKBB. This is especially true in comparison to the study from Smith et al.^12^, to which we compared our findings of the reverse MR in Supplementary Figure 2. We also observed a significant causal effect of T2D liability on non-cholesterol lipid classes, with, for example, a causal effect on increased triglycerides levels. The effects on cholesterol were not only in the negative direction, but depend on the lipid class investigated, with, for example, a positive causal estimate of T2D on VLDL-related cholesterol. We therefore argue that our observations are compatible with the dyslipidemia observed in T2D, and we acknowledge this point in lines 478-480 of the discussion. Additionally, we want to mention that we are here looking at the causal effect of T2D liability, and not of the occurrence of the disease itself.

*“[…] Overall, we observed a causal effect of T2D liability on all lipid classes, including decreases in cholesterol levels, both in HDL and LDL, and increases in triglyceride levels, characteristic of the dyslipidemia observed in T2D^11^.”*

**Interaction QTL Analysis:**

**The authors discuss effect mediation but present data on effect modification (interaction). The terminology should be corrected to accurately describe the analyses conducted.**

We thank the reviewer for this comment and apologize for the confusion as we are fully aware of the difference between interaction and mediation analyses. We have now modified the text accordingly to emphasize the difference between interaction and mediation analyses, especially in lines 20-21, 62-63, 342-344, 433.

**The interaction analysis lacks sufficient detail for proper interpretation, as only interaction coefficients are presented without the main effects. This omission hinders the interpretation of the interaction coefficients.**

**For example, ensuring that both independent variables (SNP and T2D) are oriented in the same direction concerning the risk of the investigated metabolite is crucial for inferring the interaction coefficient.**

**- Metabolite = SNP + T2D + SNP*T2D**

**It is assumed that disease status refers to prevalent T2D (not incident or any T2D). The interaction coefficient beta is interpretable only if both independents are oriented towards the same direction in predicting the outcome. Interaction effects can also be in the opposite direction, indicating that the combined effects of two risk factors are less than expected if isolated, often due to shared biology.**

We thank the reviewer for this comment which is highly pertinent. We confirm that the disease status here refers to prevalent T2D as detailed in lines 124-126 of the methods section and further added in line 218. In the revised version of the manuscript, we have added the coefficients of both independent variables (SNP and T2D) for both the discovery and replication analyses in Supplementary Table 5. For all significant interaction QTL variants, the effects of the variables SNP and T2D are in the same direction, but always in the opposite direction of the interaction variable (please see the more detailed answer to this point below). All the coefficients are in the same direction between the discovery and the replication analyses for the three variables.

**The authors claim to have identified 14 interaction QTLs, of which 40 were replicated in ESTBB. This discrepancy suggests that 40 variants were identified in the discovery analysis (as shown in the supplemental tables), with only 4 being replicated and shown in the box plots.**

In the discovery analysis, we identified 222 significant variants in the interaction analysis, corresponding to 14 metabolites. In the replication analysis, 40 interaction QTLs were identified, which map to 4 metabolites. We have now clarified this in the text in lines 344-350.

*“[…] We identified 222 variants having a significant interaction between the mQTL effect and T2D status, corresponding to 14 metabolites, including glycine and various lipids. Of these, 40 pairs of variants were replicated in the EstBB (S5 Table), covering four metabolites: percentage of free cholesterol in small HDL (S_HDL_FC_pct), percentage of phospholipids in large LDL (L_LDL_PL_pct), percentage of triglycerides in large VLDL (L_VLDL_TG_pct) and percentage of free cholesterol in large VLDL (L_VLDL_FC_pct). […]”*

**It is notable that all four replicated metabolites are percentages rather than absolute levels. For instance, the percentage of free cholesterol in small HDL shows higher levels in controls, with the T-allele increasing this metabolite, while in cases, the metabolite is lower and unaffected. Conversely, in L_LDLPL, LDL levels are higher in controls than in cases, and the interaction attenuates the T-allele effect, bringing it closer to the metabolite value in cases. Here, the interaction reduces the differences between groups rather than amplifying them.**

We thank the reviewer for this comment and for further interpreting the interaction signals observed in our study. In light of the previous comment, we indeed observe for all significant interaction QTLs an attenuation rather than an amplification of the differences between cases and controls as the direction of the interaction effect is opposite to the main effects of T2D and SNP. This confirms that the affected genetic regulation is a consequence rather than a cause of T2D, which goes in line with the MR results, and with the fact that these interaction QTLs are not associated with T2D status in the latest Type 2 Diabetes Global Genetics Initiative (T2DGGI) meta-analysis. Further work is still needed to better interpret these results, which might be due to a feedback regulation of metabolite levels following the occurrence of the disease. We have now clarified these observations in the results and in the discussion section in lines 365-373, 452-456, 514-519. We have also clarified and modified the boxplots.

*Results section: “[…] All of the interaction variants replicated in the EstBB are mQTLs for the corresponding metabolites in the overall UKBB cohort but are not associated with T2D risk in the latest T2D GWAS meta-analysis^13^. These findings suggest that the different genetic regulation of metabolite levels observed between T2D patients and controls is a consequence rather than a cause of the disease, in line with the results of the reverse MR for S_HDL_FC_pct and L_VLDL_TG_pct. This also goes in line with the observed interaction effects with the different genetic regulation of metabolites decreasing the differences in metabolite levels between cases and controls. The results remained consistent after adjustment for BMI, lipid-lowering medication or metformin medication (S6 Table).”*

*Discussion sections:*

- *“[…] Further, we have shown that T2D occurrence seems to have an impact on the genetic regulation of metabolite levels. Finally, we describe that metabolite profiles are also associated with T2D complications, though no significant causal relationship could be demonstrated in the present study.”*
- *“[…] Additionally, we observed that these interactions resulted in attenuated differences in metabolite levels between cases and controls. The decreased magnitude of genetic regulation in cases might be explained by feedback mechanisms trying to restore homeostasis equilibrium following the occurrence of the disease. Further research is needed to validate this hypothesis and to further interpret these findings, which is challenged by the fact that significant metabolites correspond to percentages and not absolute values. […]”*

**The use of percentages complicates the interpretation further**.

We agree with the reviewer that the interpretation is challenged by using percentages, a point which is now discussed in lines 517-519. However, external replication in the EBB lends confidence to the robustness of these significant metabolites.

**Minor Comments:**

**- In Figure 2, consider using red for color coding instead of yellow for better clarity.**

We thank the reviewer for the suggestion and have changed figure 2 to include red color coding instead of yellow.

Reviewer #3: This manuscript (MS) aims to elucidate the consequences of type 2 diabetes (T2D) by exploring the connections between metabolite profiles, genetic factors, and T2D risk and subsequent complications. The study investigates these relationships using data from two distinct biobanks: the UK Biobank and the Estonian Biobank. The topic is both timely and relevant, with the MS employing robust methodologies. Additionally, the MS is well-written and neat. However, it could benefit from further clarifications in certain areas.

**Mayor**

**The manuscript title could be enhanced by incorporating more details about the methodologies employed. This would provide readers with a clearer understanding of the approach and scope of the study, making the title more informative and engaging.**

We thank the reviewer for this comment and have now modified the title of the manuscript accordingly to: *“Disentangling the consequences of type 2 diabetes on targeted metabolite profiles using causal inference and interaction QTL analyses”.*

**The abstract could be improved by including more detailed information about the methodologies used to investigate the sources of the observed causal associations. Providing insights into the specific analytical approaches, data sources, and techniques employed will offer readers a clearer picture of how the study addresses its research questions and supports its findings. Additionally, the abstract mentions “lipid” classes but would benefit from specifying the particular lipid classes.**

We thank the reviewer for this suggestion, which goes in line with the comments from reviewer 1. In the revised version of the manuscript, we have rewritten the abstract to include information about the metabolite panel used, the different metabolite classes, and the number of metabolites assessed. We have also specified the data used for T2D, as well as the methods employed in our study (MR and differential level analyses for associations with complications):

*“Circulating metabolite levels have been associated with type 2 diabetes (T2D), but the extent to which T2D affects metabolite levels and their genetic regulation remains to be elucidated. In this study, we investigate the interplay between genetics, metabolomics, and T2D risk in the UK Biobank dataset using the Nightingale panel composed of 249 metabolites, 92% of which correspond to lipids (HDL, IDL, LDL, VLDL) and lipoproteins. By integrating these data with large-scale T2D GWAS from the DIAMANTE meta-analysis through Mendelian randomization analyses, we find 79 metabolites with a causal association to T2D, all spanning lipid-related classes except for Glucose and Tyrosine. Twice as many metabolites are causally affected by T2D liability, spanning almost all tested classes, including branched-chain amino acids. Secondly, using an interaction quantitative trait locus (QTL) analysis, we describe four metabolites consistently replicated in an independent dataset from the Estonian Biobank, for which genetic loci in two different genomic regions show attenuated regulation in T2D cases compared to controls. The significant variants from the interaction QTL analysis are significant QTLs for the corresponding metabolites in the general population but are not associated with T2D risk, pointing towards consequences of T2D on the genetic regulation of metabolite levels. Finally, through differential level analyses, we find 165 metabolites associated with microvascular, macrovascular, or both types of T2D complications, with only a few discriminating between complication classes. Of the 165 metabolites, 40 are not causally linked to T2D in either direction, suggesting biological mechanisms specific to the occurrence of complications. Overall, this work provides a map of the consequences of T2D on Nightingale targeted metabolite levels and on their genetic regulation, enabling a better understanding of the T2D trajectory leading to complications.”*

**The manuscript focuses on a single metabolomic measurement as the exposure variable. This approach has important implications for causal inference. Relying on just one metabolite may limit the study's ability to fully capture the complexity of metabolic pathways associated with T2D risk. It might be beneficial to discuss the implications of this limitation.**

We thank the reviewer for this comment and acknowledge this limitation of our study, which is now discussed in lines 542-546:

*“[…] Furthermore, metabolites were considered individually as exposure, which does not capture overall metabolic pathways. As expected, we observed similar causal effects of highly correlated metabolites belonging to the same class. It would be of interest to integrate metabolites together, for example through multivariate MR analyses^14^ within each metabolite class, to assess their relative contribution in shaping T2D risk. […]”*

**The MS uses the term "mediation" in reference to genetic factors. However, based on the context provided, it appears that the authors are describing an interaction effect, which is more indicative of "effect modification" rather than traditional mediation analysis. Mediation typically involves exploring the mechanism through which one variable affects another through an intermediary variable, whereas effect modification refers to how the relationship between two variables changes across different levels of a third variable. It would be beneficial for the authors to clarify this distinction to ensure that the terminology accurately reflects the analytical approach used.**

We agree with the reviewer that we did not use the right terminology to refer to our interaction analyses, while we are aware of the differences with a mediation analyses. We have now modified the text accordingly, especially in lines 20-21, 62-63, 342-344, 433.

**Please provide a rationale for retaining related samples in the analysis.**

We have not retained related samples in our analysis. As explained in the supplementary methods, we have used the QC criteria provided by the UKBB (<https://biobank.ctsu.ox.ac.uk/crystal/crystal/docs/genotyping_qc.pdf>), which identifies and excludes related individuals based on the kinship matrix. We have further specified this in line 107.

**In line 94, the term “homogeneity” is used to partially justify the exclusion of non-European samples. Please avoid using this term in contexts where there are multiple sources of genetic and non-genetic heterogeneity, even among samples of European ancestry. It is crucial to recognize that variability can still exist within genetically similar samples due to other contributing factors across all populations.**

We thank the reviewer for this useful comment and fully agree with it. We have now changed the sentence in lines 106-107 accordingly:

*“[…] To minimize potential bias in MR due to differences between the exposure and outcome datasets which could be related to having different ancestry background in the two datasets, we chose to focus on unrelated individuals of European ancestry. […]”*

***Minor***

**The size of the figures makes them difficult to read. Increasing the resolution or size of the figures would improve their clarity and readability.**

We thank the reviewer for this comment and acknowledge the difficulty in representing the results for the 249 metabolites analyzed in our study. We want to confirm to the reviewer that all the figures were checked through the PACE tool recommended by PLos Genetics to ensure they have the required resolution. To improve the readability of the circular plots (previously figure 2), which summarize a lot of information, we have decided to include them as two separate figures (one for the absolute levels, and one for metabolite ratios). Yellow colors have also been changed to red colors as suggested by another reviewer. Additionally, we have removed the extra space in the bottom panel of figure 6 (previously figure 5) to increase its readability, and we have improved the resolution of figure 4 (previously figure 3).

Figure 2: Circular plot of the forward (ForMR) and reverse (RevMR) MR analyses with inverse variance weighted (IVW) estimates from the 1^st^ metabolite set (absolute metabolite values). Metabolites having increased levels associated with T2D occurrence are presented in yellow and the ones with decreased levels in blue. Non-significant metabolites, or metabolites not replicated in the 2^nd^ metabolite set are colored in grey. Metabolites are grouped according to metabolite classes. The y-axis corresponds to the q-value (qval).

Figure 3: Circular plot of the forward (ForMR) and reverse (RevMR) MR analyses with inverse variance weighted (IVW) estimates from the 1^st^ metabolite set (metabolite ratios). Metabolites having increased levels associated with T2D occurrence are presented in yellow and the ones with decreased levels in blue. Non-significant metabolites, or metabolites not replicated in the 2^nd^ metabolite set are colored in grey. Metabolites are grouped according to metabolite classes. The y-axis corresponds to the q-value (qval).

Figure 4: Boxplot showing metabolite levels according to the genotype in T2D patients and controls (the most significant variant for each of the four metabolites replicated in the EBB are represented). The metabolite levels represented are inverse-normal transformed and adjusted for the covariates used in the interaction QTL analyses. For each tested variant (ID is in the form chr_pos_NonEffectAllele_EffectAllele), its frequency in the control and in the T2D patients’ group is provided, along with the beta and p-value of association with metabolite levels. The overall p-value of the interaction test is also given.

Figure 6: Association analyses between metabolite levels and T2D complications (microvascular ‘micro’, macrovascular ‘macro’, or both complication groups ‘micro_macro’). A: Venn diagram of the significant metabolites (meta-analysis q-values lower than 5%) across complications. B: log10(q-values) of associations between metabolite levels (separated into absolute levels and derived ratios) and each complication group. –log10(q-values) are represented for positive associations, and log10(q-values) are represented for negative associations.

**In the Results and Discussion sections, some verbs are written in the present tense. Please ensure that the tense is consistent with the rest of the manuscript, typically using past tense for describing the results and discussion of completed experiments.**

We have now harmonized the manuscript to use the past tense when describing and discussing the results.

**Please include a description of all abbreviations used in the figures to enhance readability and ensure that the figures are easily interpretable.**

We thank the reviewer for this useful suggestion and have now detailed all abbreviations in the legend corresponding to each figure (please see new legends above).

References

1.	Sudlow, C., Gallacher, J., Allen, N., Beral, V., Burton, P., Danesh, J., Downey, P., Elliott, P., Green, J., Landray, M., et al. (2015). UK biobank: an open access resource for identifying the causes of a wide range of complex diseases of middle and old age. PLoS Med *12*, e1001779. 10.1371/journal.pmed.1001779.

2.	Borges, M.C., Haycock, P.C., Zheng, J., Hemani, G., Holmes, M.V., Davey Smith, G., Hingorani, A.D., and Lawlor, D.A. (2022). Role of circulating polyunsaturated fatty acids on cardiovascular diseases risk: analysis using Mendelian randomization and fatty acid genetic association data from over 114,000 UK Biobank participants. BMC Med *20*, 210. 10.1186/s12916-022-02399-w.

3.	Mbatchou, J., Barnard, L., Backman, J., Marcketta, A., Kosmicki, J.A., Ziyatdinov, A., Benner, C., O'Dushlaine, C., Barber, M., Boutkov, B., et al. (2021). Computationally efficient whole-genome regression for quantitative and binary traits. Nat Genet *53*, 1097-1103. 10.1038/s41588-021-00870-7.

4.	Purcell, S., Neale, B., Todd-Brown, K., Thomas, L., Ferreira, M.A., Bender, D., Maller, J., Sklar, P., de Bakker, P.I., Daly, M.J., and Sham, P.C. (2007). PLINK: a tool set for whole-genome association and population-based linkage analyses. Am J Hum Genet *81*, 559-575. 10.1086/519795.

5.	Li, J., and Ji, L. (2005). Adjusting multiple testing in multilocus analyses using the eigenvalues of a correlation matrix. Heredity (Edinb) *95*, 221-227. 10.1038/sj.hdy.6800717.

6.	Laakso, M., and Fernandes Silva, L. (2023). Statins and risk of type 2 diabetes: mechanism and clinical implications. Front Endocrinol (Lausanne) *14*, 1239335. 10.3389/fendo.2023.1239335.

7.	Cholesterol Treatment Trialists' Collaboration. Electronic address, c.n.o.a.u., and Cholesterol Treatment Trialists, C. (2024). Effects of statin therapy on diagnoses of new-onset diabetes and worsening glycaemia in large-scale randomised blinded statin trials: an individual participant data meta-analysis. Lancet Diabetes Endocrinol *12*, 306-319. 10.1016/S2213-8587(24)00040-8.

8.	Khankari, N.K., Keaton, J.M., Walker, V.M., Lee, K.M., Shuey, M.M., Clarke, S.L., Heberer, K.R., Miller, D.R., Reaven, P.D., Lynch, J.A., et al. (2022). Using Mendelian randomisation to identify opportunities for type 2 diabetes prevention by repurposing medications used for lipid management. EBioMedicine *80*, 104038. 10.1016/j.ebiom.2022.104038.

9.	Smit, R.A.J., Trompet, S., Leong, A., Goodarzi, M.O., Postmus, I., Warren, H., Theusch, E., Barnes, M.R., Arsenault, B.J., Li, X., et al. (2020). Statin-induced LDL cholesterol response and type 2 diabetes: a bidirectional two-sample Mendelian randomization study. Pharmacogenomics J *20*, 462-470. 10.1038/s41397-019-0125-x.

10.	Hemani, G., Zheng, J., Elsworth, B., Wade, K.H., Haberland, V., Baird, D., Laurin, C., Burgess, S., Bowden, J., Langdon, R., et al. (2018). The MR-Base platform supports systematic causal inference across the human phenome. Elife *7*. 10.7554/eLife.34408.

11.	Higuchi, S., Izquierdo, M.C., and Haeusler, R.A. (2018). Unexplained reciprocal regulation of diabetes and lipoproteins. Curr Opin Lipidol *29*, 186-193. 10.1097/MOL.0000000000000521.

12.	Smith, M.L., Bull, C.J., Holmes, M.V., Davey Smith, G., Sanderson, E., Anderson, E.L., and Bell, J.A. (2023). Distinct metabolic features of genetic liability to type 2 diabetes and coronary artery disease: a reverse Mendelian randomization study. EBioMedicine *90*, 104503. 10.1016/j.ebiom.2023.104503.

13.	Suzuki, K., Hatzikotoulas, K., Southam, L., Taylor, H.J., Yin, X., Lorenz, K.M., Mandla, R., Huerta-Chagoya, A., Melloni, G.E.M., Kanoni, S., et al. (2024). Genetic drivers of heterogeneity in type 2 diabetes pathophysiology. Nature. 10.1038/s41586-024-07019-6.

14.	Sanderson, E., Spiller, W., and Bowden, J. (2021). Testing and correcting for weak and pleiotropic instruments in two-sample multivariable Mendelian randomization. Stat Med *40*, 5434-5452. 10.1002/sim.9133.
